# Antibiotic-Free Hypothermic Storage of Boar Semen at 5 °C with Next-Day On-Farm Cooling: Fertility and Biosafety Under Field Conditions

**DOI:** 10.3390/antibiotics15050490

**Published:** 2026-05-12

**Authors:** Florian Reckinger, Anne-Marie Luther, Thu Quynh Nguyen, Anja Riesenbeck, Dagmar Waberski

**Affiliations:** 1 Unit for Reproductive Medicine/Clinic for Swine and Small Ruminants, University of Veterinary Medicine Hannover, Foundation, Bünteweg 15, D-30559 Hanover, Germany; florian.reckinger@tiho-hannover.de (F.R.); anne-marie.luther@tiho-hannover.de (A.-M.L.); thu.quynh.nguyen@tiho-hannover.de (T.Q.N.); 2 GFS Genossenschaft zur Förderung der Schweinehaltung eG, Zum Pöpping 29, D-59387 Ascheberg, Germany; riesenbeck@gfs-topgenetik.de

**Keywords:** boar semen preservation, *Serratia marcescens*, *Klebsiella oxytoca*, hypothermic semen storage, antimicrobial resistance, antibiotics, semen chilling, pig reproduction, artificial insemination

## Abstract

**Background**: Antibiotic-free approaches to boar semen preservation are gaining importance to counter emerging antimicrobial resistance. Hypothermic storage at 5 °C, instead of the conventional 17 °C, is a promising strategy to eliminate antibiotics still commonly used in extenders. For practical adoption, the method must be simple and compatible with on-farm routines. **Objective:** To assess fertility when cooling was initiated on farm one day after delivery, and to evaluate the robustness of cold-stored semen to temporary warming and subsequent re-cooling, mimicking typical handling on insemination days. **Methods**: Individual ejaculates (n = 34) from six boars were extended in Androstar^®^ Premium either without antibiotics (5 °C) or with gentamicin (17 °C control). One day after collection, antibiotic-free doses were cooled on farm to 5 °C and used alongside controls in routine insemination of 270 sows. Sperm quality was evaluated by computer-assisted semen analysis and flow cytometry, and bacterial counts were monitored. In a separate test, cold-stored doses were exposed to 20 °C for 60 min and re-cooled to 5 °C. **Results**: Farrowing rates and litter sizes did not differ between groups (*p* > 0.05). In antibiotic-free samples after 120 h, bacterial counts were mostly not detectable or low (<10^2^ CFU/mL). Sperm motility and plasma membrane integrity in cold-stored doses remained >80%, comparable to controls (*p* > 0.05). Temporary warming did not affect sperm quality or bacterial counts. **Conclusions**: Antibiotic-free semen storage at 5 °C is easy to implement in practice and maintains fertility under field conditions. Broader validation under routine conditions is encouraged in support of the One Health concept.

## 1. Introduction

Artificial insemination (AI) is the principal assisted reproduction technology in pigs and other farm animals. Since the introduction of AI into animal breeding about 60 years ago, antibiotics have been regarded as indispensable components of semen preservation media [1]. Semen inevitably contains bacteria, originating from the male microbiome and from the environment in housing and laboratory settings. Unlike semen of other species, boar semen is stored unfrozen at a comparatively high temperature, about 16–18 °C [2], because boar sperm membranes are highly susceptible to cold shock, presumably due to their low cholesterol content [3]. Effective antimicrobial control in extended semen is therefore paramount. As a consequence of the long-term, intensive use of various antibiotic classes in semen extenders, antimicrobial resistance has emerged, frequently associated with sperm damage and risks to sow health and the environment [4]. In recognition of this threat and in line with recent European Union policies restricting prophylactic antibiotic use in livestock farming, novel antibiotic-free semen preservation techniques are being developed [5]. Following the One Health framework, strategies to combat antimicrobial resistance and to curb the overuse of antibiotics are prioritized not only in human health but also in veterinary medicine and animal husbandry [6,7]. Additionally, pig farmers in particular face strong economic pressures due to the global market situation [8] and are subject to regulations and extensive documentation requirements aimed at minimizing antibiotic use.

Referring to the development of antibiotic-free alternatives in pig reproduction, hypothermic semen storage is at an advanced stage of testing. Storage of boar semen at 5 °C efficiently inhibits bacterial growth [9,10,11,12], while maintaining sperm functionality—including calcium homeostasis [13,14] and DNA integrity [9]. High fertility potential of cold-stored semen has been demonstrated in vitro [15,16] and in vivo [9,13,17].

Field studies with antibiotic-free, cold-stored semen were first reported in South America using post-cervical insemination [9,13,18]. Until 2023, EU insemination trials continued to include antibiotics due to prevailing legislation. Accordingly, in the first field trial with cold-stored semen conducted in Germany, antibiotics were still included in the extender. This trial, initially using intracervical AI, essentially confirmed the high fertility of cold-stored semen compared with conventionally preserved semen at 17 °C [17]. However, evidence for high fertility using antibiotic-free 5 °C-stored semen with traditional intracervical insemination under routine farm conditions is still lacking.

Beyond encouraging fertility outcomes, successful transfer into field conditions must consider ease of use in AI centers and on sow farms. To meet operational requirements, the original cooling protocol—controlled chilling on the day of collection [19]—was modified to include a 16–24 h holding period at 17 °C [20]. This adaptation allows AI centers to produce doses intended for 5 °C storage without changing conventional workflows, storage, or transport logistics. To date, fertility data for antibiotic-free semen subjected to delayed on-farm cooling are unavailable.

Against this background, the primary objective of this study was to evaluate sperm quality, microbiological status, and in vivo fertility of antibiotic-free semen shipped at 17 °C and cooled the next day to 5 °C on a German farm under field conditions. We hypothesized that, under these conditions, antibiotic-free semen would preserve high sperm quality and inhibit bacterial growth, thereby maintaining high fertility. A second objective was to analyze sperm quality and bacterial counts in stored doses that were returned to the semen storage unit after temporary exposure to barn room temperature, which is a common practice on large sow farms. Given the greater temperature differential between 5 °C and barn temperature compared with the conventional 17 °C storage regime, this scenario warranted investigation. We tested the hypothesis that short-term (60 min) exposure of semen doses to 20 °C does not affect sperm quality or microbiological safety. Overall, our aim was to test the practicality of antibiotic-free hypothermic semen storage in a representative European pig-breeding setting, thereby paving the way for broader implementation in practice.

## 2. Results

### 2.1. Experiment 1: Insemination Trial with Antibiotic-Free Semen Cooled on Farm

#### 2.1.1. Fertility

**Figure 1:**
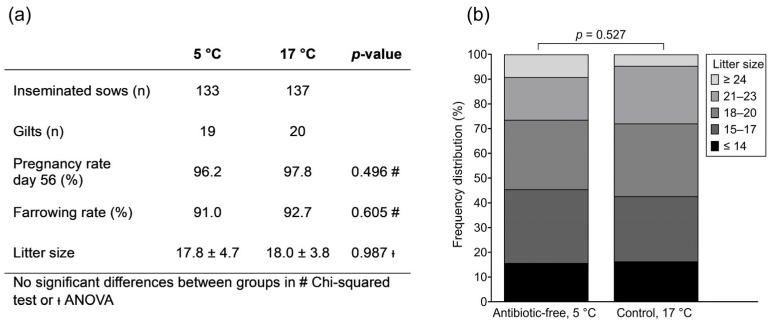
On-farm fertility after insemination with antibiotic-free, cold-stored semen (**a**). Values are presented as mean ± SD. Frequency distribution of litter size (**b**). Stacked columns represent the percentage of litters (n = 248) in each total born litter size category.

Four sows were excluded due to a heat-stress event followed by illness or death shortly before farrowing. Fertility data from 270 sows are shown in Figure 1. Parities, including gilts (n = 39), were evenly distributed between the 5 °C and 17 °C groups. Pregnancy rates and farrowing rates did not differ between groups (*p* > 0.05). In total, 248 sows farrowed 4476 piglets, with no significant difference between the two insemination groups (*p* > 0.05). Likewise, the frequency distribution of litter-size classes did not differ (Figure 1).

#### 2.1.2. Spermatology

**Figure 2:**
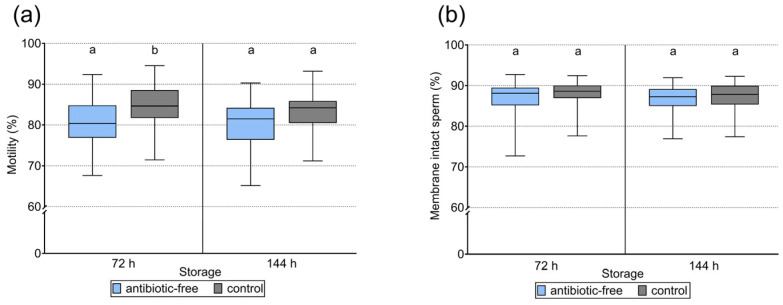
Sperm motility (**a**) and membrane integrity (**b**) in semen doses used for on-farm artificial insemination (n = 34 ejaculates). Antibiotic-free doses were cooled on farm and subsequently stored at 5 °C, whereas control doses contained gentamicin and were stored at 17 °C. Different lowercase letters indicate significant differences within a given time point (ANOVA, *p* < 0.05).

Sperm motility of the 34 semen samples used in the insemination trial is shown in Figure 2a, and kinematic data are presented in Table 1. At 72 h, total motility was higher in the 17 °C group (84.3 ± 5.5%) than in the 5 °C group (80.8 ± 6.0%; *p* = 0.046). At 144 h, total motility did not differ between 17 °C (83.2 ± 4.8%) and 5 °C (80.1 ± 5.5%; *p* = 0.952). In the 5 °C group at 144 h, the lowest motility observed was 65% in one sample; all other values were higher. At 72 h, VCL and LIN were higher in the 17 °C than in the 5 °C group (*p* = 0.015 and *p* < 0.001, respectively), whereas BCF and ALH did not differ between groups (*p* > 0.05).

Sperm membrane data are shown in Figure 2b. The proportion of spermatozoa with intact plasma membranes and intact acrosomes did not differ between temperature groups (*p* = 0.662). At 144 h, 86.8 ± 3.3% (5 °C) and 87.5 ± 3.8% (17 °C) of spermatozoa exhibited intact plasma membranes and acrosomes. In the 5 °C group, the proportion of membrane-intact sperm ranged from 72.7% to 92.7%; in the 17 °C group, from 77.4% to 92.4%.

#### 2.1.3. Microbiology

**Figure 3:**
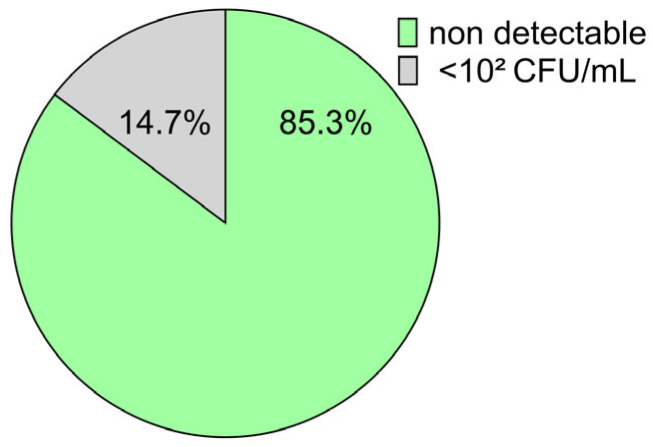
Bacterial counts in antibiotic-free semen doses stored at 5 °C for 120 h (n = 34). Most samples (n = 29) were below the detection limit (<10 CFU/mL).

The distribution of bacterial counts in semen samples stored at 5 °C for 120 h is shown as a pie chart in Figure 3. After culture on blood agar for 24 h at 37 °C, counts were <10^2^ CFU/mL in all samples. In 85.3% of samples, counts were below the detection limit (10 CFU/mL). The highest count observed was 8 × 10^1^ CFU/mL in one sample.

### 2.2. Experiment 2: Sperm Quality of Cold-Stored Semen After Temporary Warming

#### 2.2.1. Spermatology

**Figure 4:**
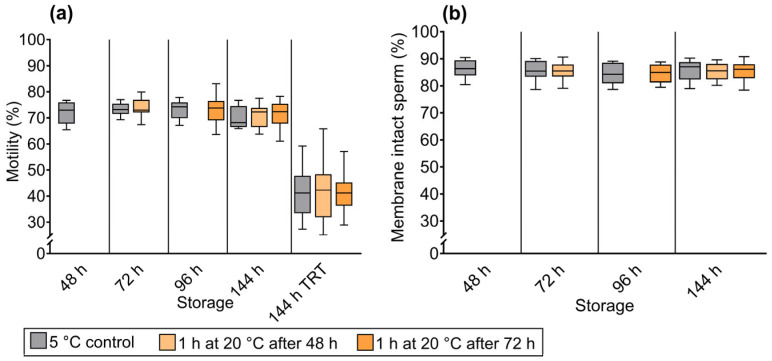
Experiment 2. Total sperm motility in doses removed from 5 °C storage for 60 min at 20 °C after 48 h or 72 h to simulate on-farm handling, then returned to 5 °C. Motility was assessed on the following day and at 144 h. At 144 h, a thermoresistance test (TRT) was performed after 3 h of incubation at 38 °C. No significant differences (*p* > 0.05) between warmed doses and controls were observed at any time point (**a**). Percentage of membrane-intact sperm (FITC-PNA negative/PI negative), showing no significant differences (*p* > 0.05) between semen dose groups (**b**).

Total motility of cold-stored semen was not affected by temporary warming for 60 min at 20 °C compared with doses held continuously at 5 °C, whether warming occurred at 48 h (*p* = 0.530) or 72 h of storage (*p* = 0.966; Figure 4a). After 144 h, mean motility ranged from 69.9% to 71.5% across treatments and controls, with no significant differences (*p* = 0.144 and *p* = 0.392). Similarly, thermoresistance testing through motility after 300 min at 38 °C following 144 h of storage showed no differences among groups.

Plasma membrane and acrosome integrity were not influenced by temporary warming for 60 min at 20 °C, regardless of whether warming occurred at 48 h (*p* = 0.754) or 72 h (*p* = 0.928; Figure 4b). After 144 h, the proportion of membrane-intact sperm was 85.3 ± 2.9% in samples warmed at 48 h (*p* = 0.525 vs. control) and 85.6 ± 3.4% in samples warmed at 72 h *p* = 0.921 vs. control), compared with 85.5 ± 3.7% in controls.

#### 2.2.2. Microbiology

Bacterial counts in raw and stored extended semen are shown in Table 2. In raw semen, counts were 1.15 × 10^4^ ± 1.34 × 10^4^ CFU/mL (mean ± SD). In extended semen stored for 48 h, counts were below the detection limit (10 CFU/mL) in both control samples and samples exposed to 20 °C. At 72 h, 1 of 6 control samples and 2 of 6 warmed samples had 10 CFU/mL; all other samples showed no detectable colonies.

## 3. Discussion

This study demonstrates that antibiotic-free hypothermic semen storage is reliable and readily applicable for both AI centers and farmers when using a simplified, on-farm cooling protocol. In line with previous reports [9,13], we found no difference between 5 °C storage without antibiotics and 17 °C storage with antibiotics in farrowing rate or litter size. Unlike earlier field trials, we employed an updated cooling procedure with one-day delayed cooling [20] and used the intracervical insemination technique.

The primary rationale for hypothermic preservation is its effective inhibition of bacterial growth, including drug-resistant, fast-growing species such as *Serratia marcescens* and *Klebsiella oxytoca* [11,12]. Delayed cooling from 17 °C to 5 °C was recently introduced [20] to facilitate logistics in AI centers. The present field data show that when doses were held at 17 °C without antibiotics for one day, bacterial growth remained efficiently inhibited during subsequent long-term storage at 5 °C, thus confirming previous in vitro data [12,21]. We used unspiked ejaculates with their natural bacterial load. Notably, antimicrobial efficacy against *Serratia* spp. is also maintained when doses are cooled the day after collection [20]. Thus, in settings where effective antibiotics are unavailable, hypothermic storage is currently the only practical means to suppress the growth of highly spermicidal bacteria—at least until contamination sources are eliminated. Using antibiotic-free doses at 17 °C on the first day before chilling is safe because bacterial proliferation, even of *Serratia* spp., has not yet entered the exponential growth phase [22]. Although an extended holding period at 15 °C for 48 h before cooling has even been associated with higher in vitro fertilization results than cooling on day 1 [16], this approach could be harmful if drug-resistant, fast proliferating bacteria are present.

From a spermatological perspective, delayed cooling is advantageous compared with the original protocol, provided that the cooling rate from 17 °C to 5 °C remains within ~0.02–0.04 °C/min [20]. Controlled cooling to 5 °C mitigates stress associated with the thermotropic phase transition of sperm membrane lipids [3,23]. In the present trial, batches consisting of 25–40 semen tubes were placed in plastic boxes and reached 5 °C within ~6 h, corresponding to a cooling rate of ~0.03 °C/min. When smaller batches are cooled, additional insulation should be used to avoid overly rapid cooling and thereby protect boar spermatozoa from chilling injury. Consistent with prior work, holding times before cooling increase sperm resilience to chilling stress [24,25,26]. Nevertheless, a mild chilling effect was apparent at 72 h, with biological meaningless slightly lower motility than in the 17 °C control (Δ: 3.5%). By 144 h, however, motility no longer differed between storage temperatures. Together with prior observations across a range of functional parameters, this suggests an early, cooling-induced loss of a small subpopulation of fragile spermatozoa, while the remaining cells exhibit resilience to diverse stressors, including long-term storage [27], vibration exposure [28,29], thermal stress [14,15], and in vitro capacitation treatment [30]. In addition to high biosafety, the robustness of semen stored at 5 °C further supports the use of hypothermic storage, with or without antibiotics.

At the farmer’s request, six boars from the TN Tempo breeding line were used in this study. Semen stored under hypothermic conditions from all six boars met the national guidelines for the use of preserved boar semen in AI [31]. Together with previous studies showing that most individual boars, regardless of breed, are suitable for cold storage [17,32], this underscores the broad applicability of hypothermic semen preservation in practice.

The insemination dose contained 2.3 × 10^9^ spermatozoa, slightly higher than conventional counts for intracervical insemination in Germany and the Netherlands but consistent with common practice in many other countries. This setting provided a safety buffer for seasonal effects [33], particularly as the field trial occurred during summer. The lower limits of sperm numbers for hypothermically stored semen under routine conditions remain to be defined and warrant further controlled field trials.

In Experiment 2, the robustness of hypothermic preservation was confirmed in cold-stored doses briefly exposed to typical barn temperatures (18–20 °C) [7]. This scenario reflects common practice in larger sow herds, where the exact number of sows to be inseminated cannot always be predicted and unused doses are returned to the storage unit. A transient temperature excursion from 5 °C to approximately 10 °C and back to 5 °C, caused by 60 min exposure to simulated barn temperature, did not affect sperm quality or bacterial counts. Classical studies indicate that boar spermatozoa can be damaged by rapid warming (as by rapid cooling) within the 5–30 °C range [34]. In our practical simulation, warming and re-cooling occurred relatively slowly within a narrow range that lay below the membrane lipid phase transition [3]. As noted above, viable chilled sperm are generally robust against multiple stressors. In practice, warming can be further minimized by using insulated boxes in the insemination barn. From a microbiological perspective, brief warming well below the optimal growth temperature of mesophilic contaminants typical in boar semen would not be expected to promote growth, consistent with our findings.

## 4. Conclusions

This study supports the translation of antibiotic-free hypothermic semen storage into routine practice by demonstrating high fertility and biosafety with next-day, on-farm cooling to 5 °C. Shipping doses at 17 °C on the day of collection facilitates adoption in AI centers by preserving established processing and logistics. Cooling to 5 °C on farm the next day is robust and feasible. It requires no changes to insemination management, only the addition of a standard refrigerator alongside the 17 °C storage unit. The additional costs, including energy, are outweighed by the greater reliability and long-established performance of conventional refrigeration compared with the more sensitive 17 °C cabinets. Further field evaluation is encouraged, especially by pioneering pig breeders aiming to reduce antibiotic inputs and support the One Health concept. Both AI centers and their customers can benefit from the high biosafety of hypothermically preserved semen doses.

## 5. Materials and Methods

### 5.1. Chemicals and Media

Unless otherwise stated, analytical-grade chemicals were obtained from Sigma-Aldrich Productions GmbH (Steinheim, Germany). The semen extender Androstar^®^ Premium was supplied by Minitüb GmbH (Tiefenbach, Germany). Fluorescent stains were purchased from Biozol Diagnostica GmbH (Hamburg, Germany), Thermo Fisher Scientific GmbH (Schwerte, Germany), and Biomol GmbH (Hamburg, Germany). Sheep blood agar plates were sourced from Thermo Fisher Scientific GmbH (Schwerte, Germany).

### 5.2. Experiment 1: Insemination Trial with Antibiotic-Free Semen Cooled on Farm

This experiment evaluated in vivo fertility using antibiotic-free semen doses transported at 17 °C to farms, stored there overnight at 17 °C and chilled the next day on farm to 5 °C, compared with doses from the same ejaculates containing antibiotic and continuously stored at 17 °C on farm. Spermatology and microbiology data from the inseminated semen batches were evaluated up to 144 h of storage.

#### 5.2.1. Semen Collection and Processing

Semen collection and processing were conducted at an EU-authorized AI center of the GFS cooperative in Germany, integrated into the routine production process. Six healthy, sexually mature boars of the TN Tempo line were selected at the farmer’s request and pretested in vitro for suitability for hypothermic storage by assessing sperm motility and membrane integrity, as described in Section 5.4. All boars met the minimum sperm requirements for preserved semen according to the guidelines of the German Livestock Association (BRS) [31]. Boar housing, handling, and semen collection complied with animal welfare regulations under the supervision of the competent veterinary authority. Ejaculates were collected once weekly by professional technicians using the gloved-hand method and processed as individual ejaculates. After an isothermal 1:1 predilution with Androstar^®^ Premium, concentration and motility were assessed by computer-assisted sperm analysis (CASA) using an eFlow chamber (Minitüb GmbH, Tiefenbach, Germany). Normospermic ejaculates were split into two subsamples: one was extended with 0.125 mg/mL gentamicin sulfate (control), and the other was extended without antibiotics. In both groups, the final sperm concentration was 25 × 10^6^/mL in a final volume of 90 mL per dose. Gentamicin-containing doses served as 17 °C controls.

#### 5.2.2. Semen Cooling and Storage

**Figure 5:**
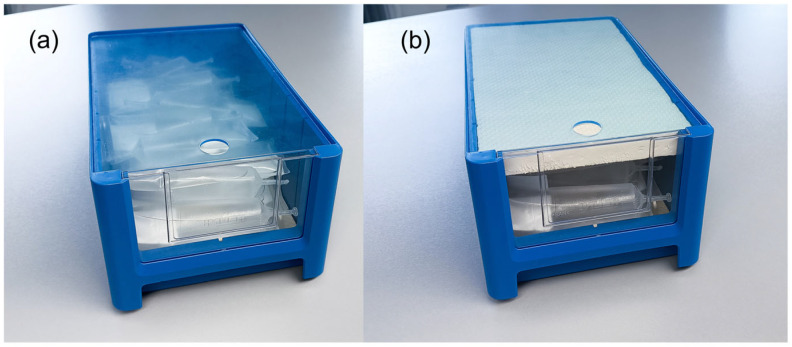
Container for storing semen doses (**a**). Semen doses in the blue container were placed in 17 °C semen storage unit upon arrival at the farm. On the following morning (22–24 h after semen collection), the entire container was transferred to a 5 °C refrigerator by farm staff. When fewer than 20 doses were present, an additional Styrofoam layer was placed on top of the box to moderate the cooling rate (**b**).

After final dilution, semen doses were shipped in temperature-controlled transport boxes set to 17 °C to a sow farm located 30 km from the AI center. All doses were stored in a temperature-controlled cabinet at 17 °C upon arrival. The next morning (18–22 h after collection), only the antibiotic-free doses were removed by farm staff from the 17 °C cabinet, placed into a plastic box, and transferred to a 5 °C refrigerator. If fewer than 20 doses were cooled, the top of the plastic box was covered with a Styrofoam layer (Figure 5) to achieve the recommended cooling curve for 5 °C storage [20]. The internal temperature of the semen tubes reached 5 °C approximately 6 h later, corresponding to a cooling rate of ~0.03 °C/min. Doses were stored until needed for insemination, for up to one week. In parallel with on-farm inseminations, samples from each boar and group were sent to the University of Veterinary Medicine Hannover and examined for sperm quality at 72 h and 144 h, and for microbiology at 120 h.

#### 5.2.3. Farm and Insemination Management

The insemination trial was conducted from mid-April to mid-June. Farm characteristics and insemination management are summarized in Table 3. The experimental conditions were aligned with the farm’s routine management. The lactation period was 28 days, with weaning on Wednesday mornings. Estrus detection and insemination were performed by farm staff. Estrus was checked twice daily in the presence of a teaser boar. Considering parity and body condition score, sows were randomly allocated to the treatment groups. Sows with a weaning-to-estrus interval >6 days and repeat breeders were excluded. Intracervical insemination was performed 12–16 h after estrus detection and repeated every 12 h until the end of standing reflex. The age of the semen at first insemination ranged from 24 h to 168 h (median 72 h). To ensure comparable estrus-to-insemination intervals and semen age across groups, semen doses from both treatment groups were used on each insemination day. Pregnancy was diagnosed by ultrasonography at three and five weeks after insemination. Fertility outcomes were recorded as pregnancy rate at day 56, farrowing rate, and litter size (total born).

### 5.3. Experiment 2: Sperm Quality of Cold-Stored Semen After Temporary Warming

This experiment tested the effect of temporarily warming cold-stored semen to ambient temperature (20 °C) for 60 min on sperm quality, simulating the return of unused doses to the storage unit after being taken to the insemination barn.

#### Semen Collection and Processing

Semen was collected at weekly intervals by experienced personnel from 12 sexually mature, fertile boars of different breeds (Piétrain, Large White, Landrace) housed at the Unit for Reproductive Medicine, University of Veterinary Medicine Hannover. The boars (2–7 years old) were housed and managed in accordance with the European Commission directive on pig welfare. All procedures were approved by the institutional Animal Welfare Committee. The ejaculates were processed as individual samples without pooling. Semen was extended to 20 × 10^6^/mL in a final volume of 100 mL, held for 24 h at 17 °C, and then cooled to 5 °C in a refrigerator [20]. After 48 h or 72 h of storage, a subset of doses was removed from the refrigerator, kept for 60 min at 20 °C, and then returned to 5 °C. Control doses were maintained continuously at 5 °C for 144 h. Semen analyses—including sperm motility, a thermoresistance test after 144 h of storage, and plasma membrane and acrosome integrity—were performed at 48 h, 72/96 h, and 144 h. Bacterial counts were measured in raw semen, immediately after the warming period before return to 5 °C, and in control samples.

### 5.4. Spermatology

#### 5.4.1. Computer-Assisted Semen Analysis

Sperm motility and kinematic parameters were assessed using computer-assisted semen analysis (CASA) with AndroVision^®^ software (version 1.2; Minitüb, Tiefenbach, Germany). Samples were incubated for 30 min at 38 °C, and 5 µL were loaded into a Leja counting chamber (Leja Products B.V., Nieuw-Vennep, The Netherlands). A minimum of 500 spermatozoa across four fields were analyzed. Spermatozoa with curvilinear velocity (VCL) > 24 µm/s and amplitude of lateral head displacement (ALH) > 1 µm were classified as motile. Spermatozoa with VCL ≥ 41 µm/s and straight-line velocity (VSL) ≥ 15 µm/s were classified as progressively motile. Additional kinematic parameters recorded were ALH, beat cross frequency (BCF), and linearity (LIN). In Experiment 2, thermoresistance was assessed by evaluating motility after 300 min of incubation at 38 °C.

#### 5.4.2. Flow Cytometry

Membrane integrity was evaluated by flow cytometry using a CytoFLEX cytometer equipped with CytExpert 2.4 software (Beckman Coulter GmbH, Krefeld, Germany) as described by [19]. Briefly, semen aliquots were stained with propidium iodide (PI; 1.0 µg/mL), fluorescein-conjugated peanut agglutinin (FITC-PNA; 0.6 µg/mL), and Hoechst 33342 (0.45 µg/mL) and incubated for 5 min at 38 °C. Fluorescence signals were recorded for 10,000 events on detectors FL-1 (450/45 nm BP), FL-2 (525/40 nm BP), and FL-3 (610/20 nm BP). Spermatozoa with intact plasma membranes and acrosomes were identified by positive Hoechst staining and negative PI and FITC-PNA signals.

### 5.5. Microbiology

Bacterial counts were determined in antibiotic-free semen samples after 120 h of storage at 5 °C (Experiment 1) and after 48 h or 72 h of storage (Experiment 2). Tenfold serial dilutions were prepared in phosphate-buffered saline (PBS) from 10^−1^ to 10^−3^. From each dilution, 100 µL were plated on Columbia agar with sheep blood and incubated aerobically at 37 °C for 24–48 h. Colonies were counted, and total bacterial numbers were calculated and expressed as colony-forming units per milliliter (CFU/mL). The detection limit was 10 CFU/mL, corresponding to one colony on the 10^−1^ dilution plate. Plates without colonies were recorded as non-detectable (n.d.).

### 5.6. Statistical Analysis

Data were analyzed using SAS Studio (Enterprise 3.81; SAS Institute Inc., Cary, NC, USA). All datasets were checked for normality and analyzed by two-way ANOVA (PROC GLM). In Experiment 1, fixed factors were semen group (antibiotic-free vs. control), time point, and their interaction. When overall effects were significant (*p* < 0.05), Tukey’s post hoc test was applied. Spermatology data are presented as mean ± standard deviation (SD). Fertility data were exported from the farm management software. The variable “litter size” was analyzed by one-way ANOVA. “Pregnancy rate,” “farrowing rate,” and the frequency distribution of litter size were analyzed using chi-squared tests (significance at *p* < 0.05). In Experiment 2, paired T-tests were used to compare treatments with controls. The significance level was set at *p* ≤ 0.05.

## Figures and Tables

**Table 1:** Sperm quality assessed in parallel with the field insemination trial. Samples from each ejaculate (n = 34) were evaluated at 72 h and 144 h. Values are presented as mean ± SD and as minimum and maximum.

	72 h		144 h	
	5 °C	17 °C	5 °C	17 °C
Progressive Motility (%) Min.–Max.	71.5 ± 9.4 ^a^ 49.9–86.0	77.0 ± 7.3 ^a^ 62.2–92.0	67.7 ± 12.5 ^a^ 38.8–85.2	73.2 ± 9.7 ^a^ 52.5–87.7
VCL (µm/s) Min.–Max.	117.7 ± 21.4 ^a^ 64.7–147.8	131.5 ± 16.3 ^b^ 102.3–162.8	128.4 ± 19.4 ^a^ 82.0–169.3	135.9 ± 17.5 ^a^ 99.1–175.6
BCF (Hz) Min.–Max.	27.3 ± 2.9 ^a^ 21.1–32.1	28.3 ± 3.0 ^a^ 22.9–34.2	26.7 ± 2.6 ^a^ 22.2–31.2	26.5 ± 3.1 ^a^ 21.5–33.7
LIN Min.–Max.	0.43 ± 0.04 ^a^ 0.35–0.51	0.47 ± 0.04 ^b^ 0.39–0.56	0.41 ± 0.05 ^a^ 0.32–0.53	0.45 ± 0.04 ^b^ 0.35–0.53
ALH (µm) Min.–Max.	0.97 ± 0.15 ^a^ 0.58–1.20	1.04 ± 0.12 ^a^ 0.82–1.28	1.05 ± 0.15 ^a^ 0.67–1.37	1.11 ± 0.15 ^a^ 0.85–1.56

Group differences within time point are marked with different superscript letters (*p* < 0.05).

**Table 2:** Experiment 2: Bacterial counts in raw semen and in extended semen immediately after 60 min at 20 °C, simulating exposure to ambient temperature when semen doses are removed from the refrigerator on the second or third day for on-farm insemination. Values are reported as CFU/mL (minimum–maximum). Non-detectable (n. d.) indicates <10 CFU/mL on agar culture.

CFU/mL	Min.	Max.
Raw semen	3 × 10^2^	3.6 × 10^4^
5 °C control	n. d.	1 × 10^1^
60 min at 20 °C after 48 h	n. d.	n. d.
60 min at 20 °C after 72 h	n. d.	1 × 10^1^

**Table 3:** Farm and insemination management.

	Farm
Boars (n)	6
Sows in production (n)	650
Sows per insemination group (n)	32
Production cycle	Weekly
Semen age at first AI (median, min-max)	72 h (24 h–168 h)
AI technique	Cervical
Number of sperm per semen tube (90 mL)	2.3 × 10^9^
Number of inseminations per estrus and sow (Mean)	2.6
Estrus control after weaning	Twice a day
First AI after detection of estrus	12 h–16 h

## Data Availability

The data presented in this study are available upon request from the corresponding author.
